# The Role of an IL-10/Hyaluronan Axis in Dermal Wound Healing

**DOI:** 10.3389/fcell.2020.00636

**Published:** 2020-07-17

**Authors:** Kavya L. Singampalli, Swathi Balaji, Xinyi Wang, Umang M. Parikh, Aditya Kaul, Jamie Gilley, Ravi K. Birla, Paul L. Bollyky, Sundeep G. Keswani

**Affiliations:** ^1^Laboratory for Regenerative Tissue Repair, Division of Pediatric Surgery, Department of Surgery, Baylor College of Medicine and Texas Children’s Hospital, Houston, TX, United States; ^2^Department of Bioengineering, Rice University, Houston, TX, United States; ^3^Medical Scientist Training Program, Baylor College of Medicine, Houston, TX, United States; ^4^Division of Neonatology, Department of Pediatrics, Texas Children’s Hospital, Houston, TX, United States; ^5^BIOLIFE4D, Houston, TX, United States; ^6^Division of Infectious Diseases, Department of Medicine, Stanford University School of Medicine, Stanford, CA, United States

**Keywords:** dermal scarring, fetal wound healing, inflammation, IL-10, extracellular matrix, hyaluronan, T lymphocytes

## Abstract

Scar formation is the typical endpoint of postnatal dermal wound healing, which affects more than 100 million individuals annually. Not only do scars cause a functional burden by reducing the biomechanical strength of skin at the site of injury, but they also significantly increase healthcare costs and impose psychosocial challenges. Though the mechanisms that dictate how dermal wounds heal are still not completely understood, they are regulated by extracellular matrix (ECM) remodeling, neovascularization, and inflammatory responses. The cytokine interleukin (IL)-10 has emerged as a key mediator of the pro- to anti-inflammatory transition that counters collagen deposition in scarring. In parallel, the high molecular weight (HMW) glycosaminoglycan hyaluronan (HA) is present in the ECM and acts in concert with IL-10 to block pro-inflammatory signals and attenuate fibrotic responses. Notably, high concentrations of both IL-10 and HMW HA are produced in early gestational fetal skin, which heals scarlessly. Since fibroblasts are responsible for collagen deposition, it is critical to determine how the concerted actions of IL-10 and HA drive their function to potentially control fibrogenesis. Beyond their independent actions, an auto-regulatory IL-10/HA axis may exist to modulate the magnitude of CD4^+^ effector T lymphocyte activation and enhance T regulatory cell function in order to reduce scarring. This review underscores the pathophysiological impact of the IL-10/HA axis as a multifaceted molecular mechanism to direct primary cell responders and regulators toward either regenerative dermal tissue repair or scarring.

## Clinical Significance

Dermal wound healing is an intricate process, driven by fibrotic mechanisms that cause scarring at the site of injury. Though the underlying mechanisms of wound healing are not fully understood, caring for wounds is a large part of our healthcare system. More than 100 million people in developed countries are estimated to develop scars annually, largely resulting from medical procedures (Bayat et al., 2003). In 2012, more than 35 million surgeries were performed in the United States and over 300 million worldwide, each resulting in at least one dermal wound. Furthermore, millions of these patients suffer from diabetes or related autoimmune disorders, leading to poor healing and chronic wounds (Weiser et al., 2016) with an estimated annual cost of care approaching $100 billion (Sen, 2019). Even in those without underlying conditions, scars reduce the biomechanical strength, elasticity, and integrity of the skin and impair its function (Scott Adzick et al., 1985; Corr and Hart, 2013). Scars also contribute to pain and psychological distress, as patients have reported feelings of anxiety regarding the appearance of the scar or the memories surrounding the instigating event (Brown et al., 2008).

The high morbidity of scar formation is not unique to the skin but is common in post-inflammatory reactions of other organs. For instance, scar tissue can be deposited in place of cardiac tissue after a myocardial infarction, which then interferes with synchronous cardiac function. Similarly, interstitial pulmonary fibrosis, a progressive condition defined by deposition of scar tissue in the lung interstitium, is caused by post-inflammatory fibrotic reactions. Renal fibrotic diseases, such as crescentic glomerulonephritis, also emphasize the systemic nature of the scar formation processes (Sziksz et al., 2015; Steen et al., 2019). Taken in a broader context, it has been postulated that fibrosis accounts for almost 50% of worldwide mortality (Wynn, 2004). Due to the high incidence and the high-risk morbidity of fibrosis, it is imperative to elucidate the mechanisms that lead to fibrosis rather than regeneration after injury so that new therapies can be developed to widely address the physical and psychosocial consequences of aberrant fibrosis.

Unlike fetal and mucosal tissues, which physiologically heal scarlessly, postnatal skin inevitably undergoes fibrotic wound healing, which begs the question of what determines the difference between regenerative and fibrotic tissue repair (Leavitt et al., 2016). In contrast to postnatal skin, fetal tissues and adult mucosal tissues feature lower inflammatory responses underscored by reduced immune cell recruitment and activation, lower transforming growth factor (TGF) -β1 levels (Whitby and Ferguson, 1991), and increased vascular maturity and keratinocyte proliferation (Glim et al., 2014; DiPietro, 2016); all of which are consistent with regenerative wound healing. Fetal skin exhibits lower infiltration of macrophages, mast cells, dendritic cells, and T lymphocytes (Adolph et al., 1993; Cowin et al., 1998; Wulff et al., 2012; Walraven et al., 2016), leading to reduced levels of pro-inflammatory interleukin (IL)-6 and IL-8 (Leung et al., 2012). On the contrary, fetal skin has elevated levels of the anti-inflammatory cytokine IL-10 (Leung et al., 2012). Moreover, fetal skin is rich in type III collagen arranged in a “basket weave” pattern, allowing it to have more elasticity, whereas adult skin has a densely packed type I collagen matrix (Kathju et al., 2012). The fetal extracellular matrix (ECM) is also uniquely characterized by increased amounts of high molecular weight (HMW) hyaluronan (HA), which has anti-inflammatory and anti-fibrotic functions. Interestingly, IL-10 has been shown to promote HMW HA synthesis in dermal fibroblasts (Balaji et al., 2017), which in turn has been shown to promote IL-10 production in lymphocytes (Bollyky et al., 2011). This suggests a possible biologic feedback loop between fibroblasts and lymphocytes, communicated by IL-10 and HMW HA, that can drive the postnatal wound healing response toward either a fibrotic, scar-forming phenotype or a regenerative phenotype.

The present review aims to address the morbidity of fibrosis as it relates to differences between tissue types and the contribution of immune cell responders to ECM remodeling, with specific notes on the role of IL-10 and HA in modulating fibroblast response to injury and the potential physiological impact of T lymphocyte driven regulation.

## Stages of Dermal Wound Healing

Dermal wound healing can be divided into four stages: hemostasis, inflammation, proliferation, and remodeling (Barnes et al., 2018; Larouche et al., 2018). The initial response to injury is the formation of a platelet plug for hemostasis, followed by the infiltration of primary immune cell responders. During the proliferative phase, granulation tissue, containing a highly vascularized network of ECM components and growth factors, is formed as keratinocytes and fibroblasts, respectively, are recruited to form an epithelial layer and deposit a new ECM. Myofibroblasts then contract and re-approximate the wound edges, and the temporary fibronectin and type III collagen matrix is replaced with a type I collagen-based scar (Rodero and Khosrotehrani, 2010). Table 1 summarizes the main cell types involved in wound healing and their functions.

**TABLE 1:** Role of various cell types in regulating healing.

**Cell type**	**Role**	**References**
Neutrophils	- Limit local infection - Secrete pro-inflammatory factors to increase immune response - Recruit leukocytes - Reduce rate of epithelialization	Devalaraja et al., 2000, Dovi et al., 2003
M1 macrophages	- Secrete pro-inflammatory cytokines - Present antigens to T cells - Associated with chronic wounds - Activate M2 macrophages late into response	Deiters et al., 2004, Ishida et al., 2008, Delavary et al., 2011, Willenborg and Eming, 2014
M2 macrophages	- Scavenge cellular debris - Increase extracellular matrix (ECM) synthesis - Secrete transforming growth factor (TGF)-β1 to recruit fibroblasts - Secrete interleukin (IL)-10 - Secrete VEGF for angiogenesis - Accelerate wound closure	Kovacs and DiPietro, 1994, Gordon, 2003, Lucas et al., 2010, Rodero and Khosrotehrani, 2010, Kathju et al., 2012, Willenborg and Eming, 2014, Okizaki et al., 2015
Fibroblasts	- Form ECM at injury site - Secrete growth factors, including TGF-β - Deposit collagen - Secrete hyaluronan (HA)	Gabbiani, 2003, Wang et al., 2011, Wight and Potter-Perigo, 2011, Rayahin et al., 2015, Avery et al., 2018, Barnes et al., 2018
Myofibroblasts	- Contract wound by applying tensile strength to ECM components - Promote type I collagen deposition	Gabbiani, 2003
T lymphocytes	- Effector T cells - Increase inflammatory cytokines, target damaged cells - CD4^+^ T cells - Release anti-inflammatory cytokines, including IL-10, and promote macrophage M2 polarization - Prevent macrophage / neutrophil infiltration - Increase microvascularization - Tregs - Dampen immune response - Reduce macrophage M1 polarization - Tr1 cells - Secrete IL-10	Gawronska-Kozak et al., 2006, Roncarolo et al., 2006, Bollyky et al., 2011, Zaiss et al., 2013, 2015, Strbo et al., 2014, Weirather et al., 2014, Arpaia et al., 2015, Lei et al., 2015, Mariani et al., 2019, Wang et al., 2019
B lymphocytes	- Produce humoral antibody response - Activate T cell response - Accelerate wound closure in chronic wounds - Associated with slowed wound closure in severe combined immune deficient (SCID) mice	Strbo et al., 2014, Sîrbulescu et al., 2017, Wang et al., 2019

Scarring is dictated by both the inflammatory and proliferative phases; since the skin plays a major role as a barrier against external pathogens, robust immune cell recruitment is necessary to prevent infection. However, inflammation also triggers fibrosis — the hallmark of the proliferative phase (Wick et al., 2013). Much of the cutting-edge research in the field focuses on the two main players in scarring: inflammation and ECM remodeling. By reciprocally regulating each other’s signaling, inflammatory reactions and ECM deposition can direct the cells at the site of injury to mold the structure of newly formed tissue.

## Inflammatory Response in Wound Healing

### Cellular Response

Upon injury, much of the pro-inflammatory environment is created by the innate immune response, which is crucial to fight potential infection and activate wound healing. The initial response is dominated by neutrophils for the first 1–2 days in order to limit infection (Kim et al., 2008). This is an important stage for increasing the secretion of inflammatory factors along with the recruitment of other leukocytes against target pathogens (Devalaraja et al., 2000). Interestingly, data from neutrophil depleted mice revealed accelerated epithelialization, suggesting that neutrophils slow wound healing. However, neutrophils do not appear to directly affect the dermal structure and collagen deposition (Dovi et al., 2003), emphasizing the multifactorial nature of scarring.

Other primary immune cell responders involved in fibrogenic wound healing include macrophages (Lucas et al., 2010; Barnes et al., 2018; Larouche et al., 2018), which secrete cytokines to drive cell recruitment and differentiation and scavenge cell debris at the site of injury via phagocytosis. The actions of macrophages are necessary for angiogenesis, granulation tissue formation, and rapid would closure during the early phases of healing (Mirza et al., 2009). Furthermore, phagocytosis by macrophages upholds the transition of the wound into an anti-inflammatory environment focused on stabilizing the matrix to prevent further damage (Wick et al., 2013). The plasticity of macrophages has been noted by their capability to differentiate into distinct subsets mainly represented by active pro-inflammatory macrophages (M1) seen early at the site of injury and anti-inflammatory macrophages (M2) seen later at the recovery stage (Gordon, 2003; Willenborg and Eming, 2014; Dal-Secco et al., 2015; Chuang et al., 2016). While M1 macrophages are activated by bacterial products, such as lipopolysaccharide (LPS) or inflammatory cytokines (Delavary et al., 2011), M1 to M2 polarization has been mainly attributed to the anti-inflammatory cytokines IL-10 and IL-4. The cytoprotective significance of these molecules is supported by evidence showing that M1 to M2 macrophage transition was delayed when cytokine function was blocked, contributing to delayed or absent collagen redistribution (Dal-Secco et al., 2015).

As stated previously, M1 macrophages are important in propagating the inflammatory response and removing harmful products resulting from skin damage, such as bacterial components and debris from damaged cells. By acting as antigen presenting cells, macrophages can also activate adaptive immunity via T lymphocyte differentiation and function (Delavary et al., 2011). Later during the proliferative phase, M1 macrophages undergo M2 polarization, initiating anti-inflammatory cytokine secretion, prominently IL-10 (Willenborg and Eming, 2014). This allows wounds to heal without inflammation-induced damage and prevents late hemorrhaging (Lucas et al., 2010). The M1 pro- to M2 anti-inflammatory transition is triggered by the phagocytosis of cellular debris and fibroblast activation (Leibovich and Ross, 1975). Specifically, the M2c subset plays an important role in removing cellular debris by upregulating cell surface receptors, stimulating ECM synthesis, and promoting angiogenesis (Rodero and Khosrotehrani, 2010; Willenborg and Eming, 2014). In that context, M2 macrophages release growth factors, including vascular endothelial growth factor (VEGF) and TGF-β1, that are important for neovascularization and granulation tissue formation (Gordon, 2003; Lucas et al., 2010; Kathju et al., 2012). These cytokines recruit both keratinocytes and fibroblasts, upregulate collagen deposition, and elicit pro-fibrotic healing (Kovacs and DiPietro, 1994; Delavary et al., 2011).

Macrophages have been shown to impact the healing of chronic wounds as evidenced by their depletion delaying fibroblast infiltration and collagen deposition, ultimately holding back wound closure (Ishida et al., 2008; Rodero and Khosrotehrani, 2010). Concomitantly, macrophages can accelerate wound closure by further differentiating into fibroblasts to enhance the production of ECM components (Sinha et al., 2018), a phenomenon also observed in chronic wounds of diabetic mice. Prolonged inflammation and lack of M1 to M2 macrophage conversion is characteristic of chronic wounds (Mirza and Koh, 2011), where the reduced expression of M2 genes and slowed wound closure can be rescued by the exogenous addition of TGF-β1 (Okizaki et al., 2015). The presence of factors that specifically recruit and activate macrophages, such as macrophage chemoattractant protein (MCP)-1, were linked to faster wound epithelialization in diabetic mice (Deiters et al., 2004; Niebuhr et al., 2008). This supports the notion that both recruitment and effective M1 to M2 macrophage polarization can impact the formation of new tissue and wound closure.

Differences between adult and fetal skin macrophage levels provide insight into their role in fibrosis. Fetal skin was shown to have fewer inflammatory cells overall and contained a greater percentage of M2 macrophages relative to adult skin (Walraven et al., 2016). This is also the case in both healthy and wounded oral mucosal tissue (Glim et al., 2015). Another difference is the type of TGF-β secreted by macrophages. Fetal skin has higher levels of TGF-β3, which is associated with reduced collagen deposition and scarless healing (Bullard et al., 2003). By contrast, TGF-β1 is the main type present in adult tissues and is a major mediator of scarring. Furthermore, the introduction of TGF-β1 to fetal skin leads to fibrosis, whereas its depletion from adult skin prevents fibrosis (Shah et al., 1995; Sullivan et al., 1995).

As demonstrated by these studies, the temporal localization of various immune cell types to a wound can impact healing by instigating an inflammatory or anti-inflammatory microenvironment. In addition to the recruitment of other responders, one of the main roles of these immune cells is the secretion of cytokines, which play an integral role in determining the presence and extent of scarring.

### Cytokine Response

The importance of inflammation at the site of injury has led to a focus on better understanding the regulation of regenerative healing by cytokines. During the initial response, two of the cytokines responsible for propagating inflammation are IL-6 and IL-8, which have been directly correlated to an increased number of macrophages in the wound (Avazi et al., 2019). In the presence of IL-6 and IL-8, both fibroblasts and epithelial cells have a diminished migratory capacity and secrete increased amounts of pro-inflammatory cytokines, including tumor necrosis factor (TNF)-α (Basso et al., 2016). Conversely, the relative lack of an inflammatory environment in their absence contributes to the phenotype of scarless healing. In contrast to adult wounds where IL-6 and IL-8 are dominant and present for longer periods after injury (Liechty et al., 1998, 2000a), fetal skin has significantly lower levels of these inflammatory cytokines (Leung et al., 2012). Strikingly, the application of IL-6 to fetal wounds causes scarring in this naturally scarless environment (Liechty et al., 2000a).

Just as pro-inflammatory cytokines are linked to fibrosis, the opposite is also true of the protective activity of anti-inflammatory cytokines against fibrosis. Namely, IL-10 is an anti-inflammatory cytokine that consistently demonstrates to be a major mediator in preventing scars (Gordon et al., 2008; Peranteau et al., 2008). The function of IL-10 was initially studied in T cell differentiation, as it is released by the helper T lymphocyte (Th2) subset to counter pro-inflammatory cytokines secreted by the Th1 subset. It has therefore been associated with diseases caused by a presence of Th2 cells, including allergies and asthma (Delavary et al., 2011). IL-10 is ubiquitously expressed and secreted by many cell types in addition to lymphocytes, including granulocytes (such as neutrophils), endothelial cells, keratinocytes, and mast cells. Expression by these alternate cell types can in turn influence both the innate and adaptive immune responses (Moore et al., 2001). IL-10 also plays a role in preventing chronic inflammatory diseases, such as ulcerative colitis and Crohn’s disease. Mutations in IL-10 or the IL-10 receptor have been associated with severe enterocolitis that originates from altered hematopoietic stem cell signaling and loss of regulatory T cell function, which leads to hyper-active effector CD4^+^ cell responses (Glocker et al., 2011; Shouval et al., 2014). The cytokine has also been considered as a therapeutic option for chronic Crohn’s disease and psoriasis and for acute encephalitis and other central nervous system diseases, as it has been shown to be effective in reducing inflammation (O’Garra et al., 2008).

IL-10 exerts its anti-inflammatory actions by transducing signals through a tetramer cell surface receptor composed of the IL-10Rα and IL-10Rβ subunits (Shi et al., 2014). Once bound, downstream signaling goes through the janus kinase - signal transducer and activator of transcription (JAK-STAT) pathways, which ultimately leads to the phosphorylation and activation of the STAT3 transcription factor (Kotenko et al., 1997; Walter, 2014; Sziksz et al., 2015). This pathway is an important gatekeeper as IL-6 also in part signals via STAT3. The balance of these factors influences the pro or anti-inflammatory cytokine milieu (Ouyang et al., 2011). IL-10 also reduces the infiltration of immune cell responders via inhibition of p38 MAPK and deactivation of HuR, an mRNA stabilizer (Rajasingh et al., 2006). For example, in a myocardial infarction model, IL-10 destabilizes mRNA encoding TNF-α, leading to less harmful left ventricular remodeling and reduced apoptosis of cardiac cells after injury (Krishnamurthy et al., 2009). Current research posits a role for IL-10 in mediating the metabolism of other cell types. For example, IL-10 induces macrophages to transition their metabolism from glycolysis to oxidative phosphorylation (Ip et al., 2017), which can drive M2 macrophage polarization (Galván-Peña and O’Neill, 2014). In the context of wound healing, this transition can mitigate M1 macrophage subset-driven inflammation at the injury site and promote effective wound healing (Willenborg and Eming, 2014).

IL-10 also facilitates the transition from the inflammatory to the proliferative phase by modulating the type and number of primary immune cell responders that migrate to a site of injury and regulating the expression of cytokines (Willenborg and Eming, 2014). The overexpression of IL-10 reduces the expression of pro-inflammatory mediators such as IL-6, MCP-1, and heat shock protein 47 (Peranteau et al., 2008). When given prophylactically, IL-10 decreases the number of pro-inflammatory cells at the wound site (Gordon et al., 2008; Peranteau et al., 2008), while concomitantly acting on macrophages to prevent the expression of inflammatory signals, such as TNF-α (Bogdan et al., 1991).

Evidence that IL-10 plays a role in ECM remodeling during injury stems from studies that show how scarring is caused by the transformation of fibroblasts to myofibroblasts, the deposition of collagen, and the reduction in matrix metalloprotease (MMP) activity. In these studies, IL-10 exerts a protective action against scar tissue formation by downregulating collagen production (Wangoo et al., 2003). This is true in different types of tissue, including models of pulmonary fibrosis and myocardial infarction (Nakagome et al., 2006; Rajasingh et al., 2006), in which IL-10 influences proteolytic enzymes to lyse the ECM and decrease macrophage TGF-β1 expression to prevent fibrosis (Nakagome et al., 2006; Shi et al., 2013, 2014).

**FIGURE 1:**
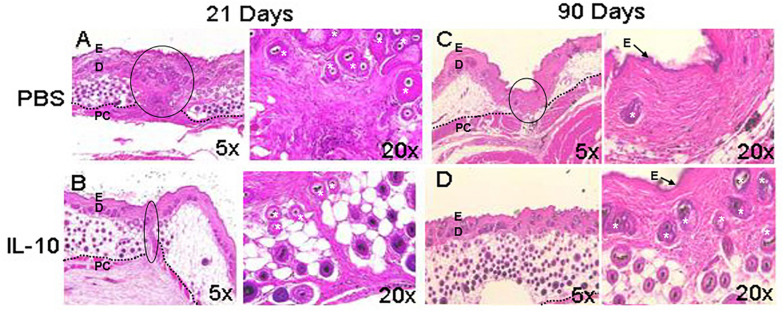
Wound healing phenotype in response to IL-10. The overexpression of interleukin (IL)-10 was accomplished using an adenoviral vector in mouse dermal wounds. Compared to PBS treated controls, IL-10 reduced scar formation. After 21 days, PBS led to the formation of a dense collagen matrix with well defined scar **(A)**, whereas IL-10 prevented formation of a defined scar **(B)**. At 90 days, the PBS treated wound shows mature scar tissue that is distinct from the surrounding skin **(C)**. IL-10 led to the generation of elements of dermal tissue, with reticular collagen and hair follicles, similar to surrounding uninjured tissue **(D)**. E, epidermis, D, dermis, *, hair follicle, PC, panniculus carnosus. The black dotted line indicates the separation between the dermis and deeper structures. The black solid line indicates scar. Images from Gordon et al. (2008), with permission.

Elevated IL-10 baseline levels are required for fetal skin to heal scarlessly, and the loss of this cytokine in fetal tissues has been associated with increased levels of inflammation in wounds and fibrotic wound repair (Liechty et al., 2000b). Accordingly, IL-10 and IL-4 deficient mice showed increased inflammatory monocytes, neutrophils, and macrophages, in conjunction with high deposition of thick collagen fibers. Treatment with IL-10 led to reduced inflammatory cell numbers and restoration of normal skin architecture and strength, which included randomly oriented collagen fibers, and clinically reduced scar size and redness (Gordon et al., 2008; Leung et al., 2012; Kieran et al., 2013; Morris et al., 2014). In support of these collective studies, Figure 1 demonstrates that overexpression of IL-10 in adult dermal wounds confers scarless healing (Gordon et al., 2008; Peranteau et al., 2008; Leung et al., 2012). IL-10 upholds this regenerative phenotype through mechanisms associated with accelerated dermal healing and by interacting with fibroblasts to modulate ECM remodeling. Specifically, IL-10 crosstalk with the ECM underscores the relevance of the interaction between inflammatory responses and ECM-driven modulation of fibrosis.

## Role of the Extracellular Matrix and Hyaluronan in Wound Healing

### Extracellular Matrix

The ECM comprises a network of structural and functional molecules that surround cells and provide a scaffold for growth and physical connectivity, which is essential to transduce environmental cues through growth factor signaling and drive inter- and intra-cellular reactions. Briefly, the ECM structure includes proteins, such as collagen fibrils, elastin, and fibronectin, which enable cell adhesion and provide tissue strength. Interspersed within the ECM are proteoglycans and glycosaminoglycans, which facilitate scaffold formation and ensure compressive strength (Briquez et al., 2015). At the core of ECM remodeling are MMPs, which play a key proteolytic role to cleave the structure at times of post-injury tissue repair (Kular et al., 2014). The ECM is also a source of growth factors and, therefore, its integrity in maintaining and releasing the required growth factors is crucial to enable healing (Briquez et al., 2015).

At the time of initial injury, the ECM scaffold is damaged, eliciting the influx of cells, cytokines, and growth factors that are necessary for the initial inflammation and repair responses. During this response, fibroblasts are stimulated by platelet derived growth factor (PDGF) and are responsible for creating a new ECM structure. A temporary ECM is built, containing fibrin, fibrinogen, and fibronectin to facilitate fibroblast adherence and secretion of proteoglycans and glycosaminoglycans (Wight and Potter-Perigo, 2011). This enhanced ECM traps inflammatory cells, thus creating a localized inflammatory microenvironment. After the formation of the granulation tissue, fibroblasts secrete growth factors, including TGF-β and fibroblast growth factor, and differentiate into myofibroblasts to contract the wound. Finally, fibroblasts remodel the provisional ECM by replacing immature type III collagen with type I collagen (Wight and Potter-Perigo, 2011; Avery et al., 2018; Barnes et al., 2018).

Differences in wound healing can be attributed to ECM composition; for instance, higher levels of type VI collagen in the lungs cause faster epithelialization than type I collagen (Mereness et al., 2018). Type VI collagen also impacts the fibroblast response in the skin by reducing the rate of fibroblast migration from the wound (Theocharidis et al., 2016). Further, the differentiation of fibroblasts into myofibroblasts is also dictated by the ECM, as high levels of fibronectin in the wound prevent early differentiation, while the expression of type I collagen later in healing induces differentiation into myofibroblasts (Avery et al., 2018). The ECM composition also differs in early gestational skin. Fetal skin, which heals scarlessly, has higher levels of type III collagen and HA, in broad contrast to adult skin where type I collagen predominates (Sawai et al., 1997; Lovvorn et al., 1999). Adult skin also has an increased overall collagen content and increased stiffness at baseline as compared to that of fetal skin (Kular et al., 2014).

Though a large percentage of healthy skin ECM is composed of type I collagen, there are significant structural differences between the ECM of healthy and scarred skin (Eming et al., 2014), which can be defined by the orientation of collagen fibers. Namely, collagen in healthy skin has a basket-weave pattern that provides high tensile strength, whereas collagen in scar tissue is arranged in thick parallel bundles (van Zuijlen et al., 2003). The collagen bundle formation in scar tissue is dependent on TGF-β1, which prevents collagen degradation and enhances the maturation of type III collagen into type I (Eming et al., 2014).

An association also exists between the tensile strength of the ECM and the forces needed to contract the wound. Specifically, fetal skin has thin collagen fibers that are under low stress, but adult skin is made up of thicker bundles of collagen that are physiologically under higher stress (Barnes et al., 2018). This causes an increased mechanical load on ECM components of adult skin, leading to decreased cell apoptosis, increased expression of cell survival genes, and hypertrophic scar formation during the proliferative healing phase (Aarabi et al., 2007). The change in phenotype is driven by immune responses, as increased mechanical forces in the wound lead to T lymphocyte activation through IL-4 and IL-13, and recruitment of macrophages and fibroblasts to the injury site (Wong et al., 2011). These results demonstrate a link between the ECM and immune responses in wound healing, and in fact, the ECM molecule HA has been identified as a central link in ECM-inflammatory cell crosstalk.

### Hyaluronan

Despite its simple disaccharide chain structure of repeating D-glucuronic acid and DN-acetylglucosamine residues, HA has multifaceted functions (Cyphert et al., 2015). Its charged structure allows it to attract large amounts of water, providing compressibility and lubrication to tissues, including joints and the skin. HA has also been shown to transduce either pro- or anti-inflammatory signals and induce or abrogate critical cell functions, including differentiation, proliferation, migration, and invasion (Cyphert et al., 2015). Which of these seemingly bipolar cues are activated depends largely on the molecular mass of HA, which can then interact with specific receptors and other ECM components to result in regeneration or fibrosis (Cyphert et al., 2015). The molecular weight of HA is determined by a complex balance of synthesis by hyaluronic acid synthase isoforms (HAS1-3) and degradation by hyaluronidase enzymes (HYAL1-4). The final HA molecular weight variants are generally classified into low molecular weight (LMW) HA (< 0.5 MDa) and HMW HA (> 1.2 MDa) variants, each with distinct effects on wound healing (Stern et al., 2006). HA with a molecular weight above 1.2 MDa (HMW HA) reduces the activity of pro-inflammatory cytokines associated with LPS and macrophage infiltration, such as IL-1α, IL-6, and TNF-α. However, when LMW HA variants were applied, increased pro-inflammatory cytokine activity was observed (McKee et al., 1996; Neumann et al., 1999). These results can potentially be explained by differences in the binding affinity of HA molecular weight variants to cognate HA receptors (CD44, RHAMM, HARE, LYVE1, layilin, TLR2, and TLR4), which could change how the multi-dimeric structures cluster at the cell membrane and transduce either pro- or anti-inflammatory signals (Hardwick et al., 1992; Carreira et al., 2001; Prevo et al., 2001; Jiang et al., 2005). However, more work is needed to elucidate the specific signaling pathways activated by LMW or HMW HA.

Injury increases the production of HA by upregulating HAS expression, while simultaneously increasing its degradation into a LMW variant by upregulating hyaluronidase expression and the presence of oxidative stress (Powell and Horton, 2005; Ruppert et al., 2014). This is important during the early inflammatory phase of wound healing, as it facilitates robust post-wounding immune responses. Prior data support a mechanism by which LMW HA acts as a damage associated molecular pattern (DAMP) and interacts with toll-like receptors (TLR) 2 and 4 on immature dendritic cells to induce the release of the inflammatory IL-1β, TNF-α, IL-6, and IL-12 cytokines. Release of these cytokines recruits neutrophils to the site of injury and drives T lymphocyte differentiation (Termeer et al., 2002; Jiang et al., 2005; Scheibner et al., 2006; Ruppert et al., 2014). Binding of LMW HA to TLR4 on antigen presenting cells also leads to dendritic maturation (Termeer et al., 2002). This signaling cascade can be enhanced by fibroblasts, which further enable the responses to DAMPs and production of IL-6, IL-8, and MCP-1 to escalate the state of inflammation (Wang et al., 2011; D’Arpa and Leung, 2017).

High molecular weight HA has shown beneficial effects during wound healing, including the reduction of inflammatory cytokine expression after ultraviolet damage of keratinocytes and the recruitment of primary immune cell responders to the site of injury after smoke inhalation (Huang et al., 2010; Hašová et al., 2011). It has been proposed that HMW HA exerts its anti-inflammatory/anti-fibrotic effects by countering the interaction of LMW HA with TLRs and preventing signaling downstream of the TLR-2 receptor (Scheibner et al., 2006). Through this process, HMW HA prevents the accumulation of advanced glycation end products, which can cause pro-inflammatory cytokine expression (Neumann et al., 1999).

The impact of HMW HA on immunity during wound healing stems from its direct interaction with primary immune cells through CD44, a receptor expressed by neutrophils, T lymphocytes, macrophages and dendritic cells (Powell and Horton, 2005). For instance, neutrophil and macrophage migration to lung tissues in the presence of LPS is significantly reduced by HMW HA/CD44 interactions (Liang et al., 2007) and the pro-inflammatory cells are more likely to undergo apoptosis when HMW HA is produced (He et al., 2013). Similarly, dendritic cells exhibit a slower maturation pattern in the presence of HMW HA, which reduces the activation of adaptive immunity (Gebe et al., 2017). In contrast to LMW HA, HMW HA directs M1 to M2 resident macrophage polarization, increasing phagocytic capacity and IL-10 expression (He et al., 2013). Remarkably, HMW HA can even reverse the M1 phenotype, reduce NOS2 and IL-12β, and increase IL-10 levels when added to LPS-activated M1 macrophages (Rayahin et al., 2015). In sum, CD44/HMW HA interactions prevent fibrotic scarring, a concept that is strongly supported by targeted *in vivo* inactivation of CD44 (KO), resulting in wounds with greater accumulation of type 1 collagen and fibrillar collagen (Govindaraju et al., 2019).

Consistent with its anti-inflammatory and anti-fibrotic effects, HMW HA is being considered as a therapeutic option to accelerate wound healing in chronic or impaired wounds. For example, application of exogenous HMW HA to diabetic wounds, which have lower baseline HA levels than healthy controls, improved healing by increasing neovascularization and TGF-β1 levels (Galeano et al., 2011). Also, the addition of HMW HA to the skin of a healthy mouse increased the rate of re-epithelialization in wound healing (Ramos-Torrecillas et al., 2015), thus supporting the benefits of HMW HA in reducing post-injury scarring. Fetal wounds have higher basal amounts of HMW HA relative to adult skin wounds, which directly prevent scarring by reducing the recruitment of fibroblasts and collagen deposition (Mast et al., 1992).

## Regulation of Fibroblasts by IL-10 and Hyaluronan

As previously discussed, both IL-10 and HMW HA can interdependently reduce pro-fibrotic wound healing, but their respective contributions to scar prevention is intertwined with their regulation of fibroblast responses to injury. Fibroblasts are the main cell type involved in new ECM production and wound closure. The myofibroblast subset, in particular, has contractile properties that can place the ECM network under stress to close the wound. In a fibrotic wound, myofibroblasts remain in the wound site for a longer period, providing them time to mature in the presence of fibronectin and TGF-β1 and synthesize α-SMA and collagen I (Gabbiani, 2003).

**FIGURE 2:**
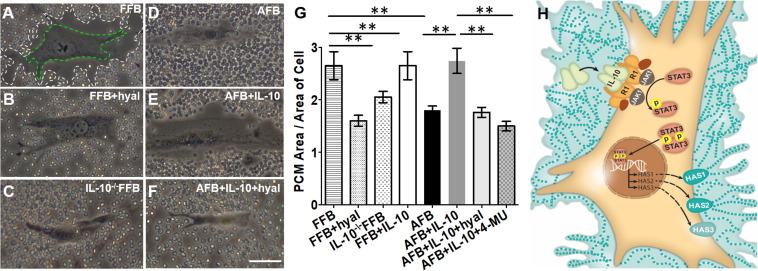
Distribution of hyaluronan (HA) in pericellular matrix (PCM) of fibroblasts in the presence of IL-10. Phase contrast imaging of fibroblasts shows differences in PCM area between fetal and adult fibroblasts. White dotted lines indicate the border of the PCM. Green dotted lines indicate the border of the cell body. Around fetal fibroblasts, there is a dense HA PCM **(A)**, which significantly decreases in the presence of hyaluronidase **(B)** or the absence of IL-10 **(C)**. In adult fibroblasts, the HA matrix is typically smaller **(D)**, but increases to a size similar to that of the fetal fibroblast in the presence of IL-10 **(E)**. This effect is reversed by the presence of hyaluronidase **(F)**. The quantification of the area of HA rich PCM relative to the area of the cell in adult and fetal fibroblasts is shown in **(G)**. **(H)** Demonstrates the pathways by which IL-10 increases the HA PCM. FFB, fetal fibroblast; AFB, adult fibroblast; HAS, hyaluronan synthase; HYAL, hyaluronidase; 4-MU, 4-methylumbelliferone. **p < 0.01; scale bar, 50 μm **(A–F)**. Figure from Balaji et al. (2017), with permission.

Since fibroblasts are responsible for forming the ECM, the regulation of their differentiation and acquired phenotypes is crucial to modulating fibrosis. Fetal fibroblasts experience slower metabolism and apoptosis than adult fibroblasts, but show increased migration and invasion (Ellis et al., 1997; Balaji et al., 2015). However, adult fibroblasts recapitulate this fetal phenotype in response to IL-10 and HA treatment in a dose dependent manner. Of note, both IL-10 and HA are required to recapitulate the migration and invasion capabilities of fetal fibroblasts in the adult counter parts. This was demonstrated when adult fibroblasts treated with IL-10 in the presence of an HA synthase inhibitor showed no improvement in migratory function (Balaji et al., 2015). IL-10 also controls the HA content of the ECM by inducing fibroblast deposition of HMW HA, which directs regenerative healing (Balaji et al., 2017). Indeed, fetal fibroblasts produce an HA rich pericellular matrix as a result of the high physiologic concentration of IL-10 in fetal tissues. A similar pattern of HA in the ECM of adult skin can be established by upregulating HAS1-3 with the addition of IL-10, as shown in Figure 2 (King et al., 2013a; Balaji et al., 2017).

STAT3, a molecule downstream of the IL-10 - IL-10R signaling, is necessary to produce high HMW HA concentrations and achieve regenerative scarless wound healing. Briefly, when IL-10 binds to its receptor, the JAK1-STAT3 pathway is activated, and STAT3 is phosphorylated to yield an active form that translocates into the nucleus and acts as a transcription factor (Riley et al., 1999; Walter, 2014). This process prevents the release of inflammatory cytokines that have been implicated in fibrosis and increases the presence of HMW HA to accelerate healing. The necessity of STAT3 in IL-10 anti-inflammatory signaling was demonstrated in STAT3 deficient macrophages, where the presence of LPS led to TNF-α production, irrespective of IL-10 treatment (Riley et al., 1999). Additionally, STAT3 is important to promote vascularization at the site of injury and enable improved healing (Krishnamurthy et al., 2009).

As previously stated, STAT3 activation is increased upon IL-10 binding to its cognate receptor (King et al., 2013a; Balaji et al., 2017). This activation proved to be beneficial in hypertrophic scars, where the addition of IL-10 similarly increases phosphorylated STAT3 (pSTAT3) and reduces expression of collagen and myofibroblast markers, including Col1, Col3, and α-SMA (Shi et al., 2014). Conversely, inhibition of IL-10 leads to lower levels of pSTAT3 and reduced nuclear localization of STAT3, which are associated with less HA production and significantly slower fetal wound healing (King et al., 2013b). These collective data support a STAT3-dependent mechanism in wound healing; namely, that STAT3 activation by IL-10 signaling induces HMW HA production and promotes vascularization to enable faster wound closure and attenuated scarring.

## Role of Lymphocytes in Wound Healing

Much of the research to date has focused on the role of inflammation, ECM, and the innate immune system in driving wound healing. Innate immune cells and their cytokine products are known to play a large role in determining whether a wound will heal via regenerative or fibrotic tissue repair. However, cells of the adaptive immune system can also drive pro- and anti-inflammatory responses to regulate the direction of post-injury wound healing (Strbo et al., 2014; Nosbaum et al., 2016; Larouche et al., 2018).

Following an acute response to injury, adaptive immunity is activated to provide antigen-specific responses. Dendritic cells are specialized cells that capture, process, and present antigens to inactive/naïve B or T lymphocytes. Upon antigen and cognate co-stimulatory signaling, B lymphocytes are activated to provide the humoral production of antigen-specific antibodies. Concurrently, T lymphocytes further differentiate into either effector CD4^+^ Th lymphocyte subsets to drive immune responses or cytotoxic CD8^+^ T lymphocyte subsets to clear aberrantly developed, damaged or infected cells. Both CD4^+^ and CD8^+^ subsets are under tight control by regulatory T cells, which modulate all immune responses (Strbo et al., 2014).

**FIGURE 3:**
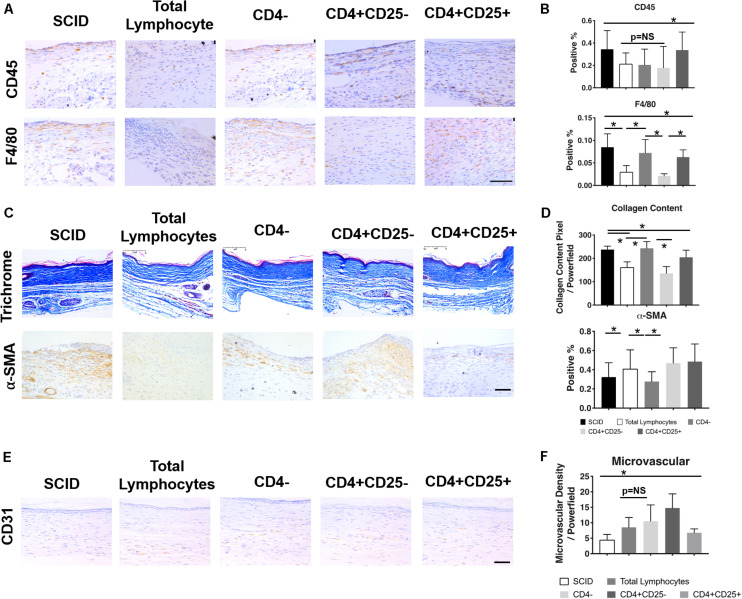
Relationship between T lymphocytes and scarring in severe combined immune deficient (SCID) mice. IHC images **(A)** and quantification **(B)** of CD45^+^ leukocytes and F4/80^+^ macrophages show changes in their levels after the introduction of total lymphocytes, CD4^–^ lymphocytes, CD4^+^CD25^–^ lymphocytes, and CD4^+^CD25^+^ lymphocytes. Similar changes with the introduction of T lymphocytes were seen in collagen content and the presence of myofibroblasts in the trichrome and α-SMA stained tissue, respectively **(C,D)**. Generally, a reduction in pro-inflammatory/pro-fibrotic cells and collagen deposition was observed after the introduction of specific T cell subsets. There was also an increase in microvascularization after the introduction of particular lymphocyte subsets **(E,F)**. *p < 0.05; scale bars, 75 μm **(A)**, 200 μm **(C,F)**. Figure from Wang et al. (2019), with permission.

While both B and T lymphocytes have roles in the development of fibrosis, the significance of T lymphocytes in wound healing can be readily demonstrated in immune deficient models such as athymic mice and severe combined immune deficient (SCID) mice. The athymic mouse models comprise a variety of immune deficient strains that lack functional mature T lymphocytes. Early studies using athymic mice highlight the importance of temporal changes in T cell levels—their early presence is necessary for restoring the biomechanical strength of skin, whereas later introduction of T lymphocytes reduces skin integrity (Barbul et al., 1989). A common denominator among most of these models is the low levels of collagen, high levels of HA, and increased tensile strength of the skin, similar to the profile seen in fetal skin (Gawronska-Kozak et al., 2006). These observations differ in SCID mice, which lack both B and T lymphocytes, where wound healing is accelerated. These mice show faster granulation tissue formation, epithelial growth, and collagen deposition; however, this leads to increased scarring. Figure 3 demonstrates how the reconstitution of lymphocytes, specifically CD4^+^ Th lymphocytes, prevents macrophage and neutrophil infiltration and collagen deposition, while increasing microvascular formation. The end result is less inflammation and fibrosis (Wang et al., 2019). On the other hand, the adoptive transfer of B cells in animal models with chronic wounds accelerates wound closure, increases fibroblast infiltration and TGF-β1 expression, and decreases cell apoptosis within the wound space (Sîrbulescu et al., 2017). Though the pathophysiological significance of B and T lymphocytes in post-injury tissue repair have not been specifically defined, the outcomes from their presence within wounds highlight the complexity and multifactorial nature of immune responses to wound healing.

The role CD4^+^ Th lymphocytes is more nuanced, as CD4^+^ cells can further differentiate into Th1 or Th2 subsets. Th1 cells are responsible for cellular immune responses while Th2 cells are important in humoral responses. There is a constant interplay between the levels of these cell types, where the cytokines secreted by one suppress the other, allowing the immune response to be targeted to a specific type of pathogen (Romagnani, 2000; Wynn, 2004). These subsets also have opposing roles in the fibrotic response (Wynn, 2004). Cytokines from the Th1 response, such as interferon-γ, create an anti-fibrotic healing profile (Wynn et al., 1995; Widney et al., 2000). However, cytokines from Th2 cells, most importantly IL-13 (Chiaramonte et al., 1999; Saito et al., 2003), accelerate wound healing, but do so at the cost of increased collagen expression and fibrosis (Kumar et al., 2002; Sandler et al., 2003). Interestingly, a predominance of Th2 cells consistently correlates with M1 to M2 macrophage polarization and Treg activation during wound healing, which strongly suggests a role for T lymphocytes in the resolution of inflammation and wound regeneration (Mariani et al., 2019). In fact, there is emerging evidence of a dynamic interplay with innate immunity supported by the observation that early neutrophil signaling contributes to subsequent Th2 influence on macrophage M2 differentiation to promote scarless tissue repair (Tacchini-Cottier et al., 2000; Chen et al., 2014; Ma et al., 2016; Mariani et al., 2019). Therefore, the balance between the cytokine profile and immune cell presence is crucial in guiding healing.

### CD4^+^ T Regulatory Lymphocytes

Regulatory T cells comprise a subset of T lymphocytes that are known to modulate the magnitude of immune responses to uphold immune tolerance and control the direction of inflammatory outcomes. Recent studies show that there are distinct subsets of regulatory cells, namely: (1) Tregs characterized by CD25^+^CD4^+^ markers that express the transcription factor Foxp3 and (2) Tr1 cells distinguished by their lack of Foxp3 expression (Pellerin et al., 2014). The presence of Foxp3 propagates a Treg response, which in turn increases tolerance to antigens by directly interacting with effector immune cells and altering cytokine profiles (Roncarolo et al., 2006; Wu et al., 2006; Zhao et al., 2006; Burzyn et al., 2013; Roh et al., 2015). Though both cell types secrete IL-10 to modulate the immune response (Vieira et al., 2004; Hossain et al., 2013), Tr1 cells are antigen specific (Gregori et al., 2010) and employ IL-10 as their primary mechanism of suppressing antigen presenting cell and T cell activity (Gregori et al., 2012).

Tregs and their anti-fibrotic properties are dependent on HA binding via its receptor CD44, which is upregulated on activated T cells and increases Treg signaling. Treg binding of HA via CD44 is associated with an upregulation of Foxp3 and lower proliferation of the immune cells typically involved in an inflammatory response (Bollyky et al., 2007). Furthermore, the increased expression of Foxp3 can act as a signal of Treg activation and the transition toward a reduced immune response (Bollyky et al., 2009). HMW HA, but not LMW HA, also induces differentiation into Tr1 cells, which regulate inflammation via IL-10 (Bollyky et al., 2011).

The capacity of Tregs to remain at the site of the wound has been attributed to their upregulation of the epithelial growth factor receptor (EGFR), which readily binds the mast cell derived ligand amphiregulin (Zaiss et al., 2013; Mariani et al., 2019), enabling access to the wounded tissue for cytoprotective T lymphocytes. Here, Tregs can expand, continue to increase the EGFR ligand, and thus induce the proliferation and differentiation of regenerative tissue repair cell mediators, including progenitor stem cells. When the wound healing response transitions away from inflammation, Tregs are recruited to reduce the presence of CD45^+^ leukocytes at the site of injury by secreting anti-inflammatory cytokines and by stimulating receptors on T cells that prevent activation. Additionally, by expressing CTLA4, Tregs reduce T lymphocyte interaction with antigen presenting cells, therefore decreasing their co-stimulation (Lei et al., 2015).

Previous studies sustain that CD4^+^ Th2 lymphocytes influence the anti-inflammatory/fibrotic M1 to M2 transition and regenerative healing, which is supported by an interactive crosstalk with regulatory cells. In this way, Tregs can become the deciding factor to direct homeostatic resolution of inflammation during post-injury tissue repair by increasing the release of anti-inflammatory IL-10 at the wound microenvironment and reducing or even abrogating fibrogenic activity. Tregs can also potentiate anti-inflammatory M1 to M2 macrophage conversion, and can persist and increase their functionality to compensate for the absence of other CD4^+^ T lymphocyte subsets (Zaiss et al., 2013, 2015; Arpaia et al., 2015; Mariani et al., 2019).

Since Tregs promote anti-inflammatory reactions, it would be expected that they would reduce fibrosis in wound healing, which has been observed in multiple tissue models (Li et al., 2018). For example, in the skin, the presence of Foxp3+ Tregs increased granulation tissue formation and the rate of wound closure (Nosbaum et al., 2016). In cardiac muscle after a myocardial infarction, the presence of Tregs prevented the differentiation of macrophages toward an M1 phenotype (Weirather et al., 2014). In lung tissue, the presence of Tregs led to reduced fibroblast recruitment to the wound (Garibaldi et al., 2013). This data supports the well-established pattern that a reduction in inflammation and maintaining the appropriate time scale for the recruitment of different cell types can improve healing and attenuate scarring.

### IL-10 Hyaluronan Axis Regulating Lymphocyte and Fibroblast Crosstalk

These observations discussed so far on inflammation and ECM responses during wound healing support the role of IL-10, HA, CD4^+^ T cells and fibroblasts in regulating fibrosis. IL-10 promotes the decrease of fibrotic cytokines and regulates the fibroblast phenotype, but also exerts its functions in an HA dependent manner. For instance, IL-10 has the capacity to induce fibroblast differentiation and function to mirror the phenotype and physiological features of fetal fibroblasts and achieve the synthesis and secretion of increased levels of HMW HA (Balaji et al., 2017). Furthermore, HMW HA stimulates CD4^+^ Th1 to Th2 lymphocyte polarization, via CD44 receptor signaling, to drive regenerative tissue repair. The HMW HA - CD44 axis can also facilitate the expansion and functional performance of the anti-inflammatory, regenerative Treg subset. Through an upregulation of Foxp3 expression, these cells reduce the presence of primary inflammatory cell responders to dermal injury (Bollyky et al., 2007). Tr1 cells also secrete IL-10, which provides positive feedback to auto-regulate the Th lymphocyte repertoire (Bollyky et al., 2011). These CD4^+^ lymphocyte subsets, such as Th2, Tr1, and Foxp3^+^ Treg lymphocytes, are in part characterized by IL-10 production. Taken together, these findings may represent the underpinnings of a potential lymphocyte-fibroblast feedback loop. That is, the loop wherein IL-10 promotes HMW HA synthesis in fibroblasts and HMW HA regulates CD4^+^ lymphocyte IL-10 expression to direct dermal wound healing outcomes. Recent data have indicated a direct crosstalk between CD4^+^ T lymphocytes and fibroblasts to regulate ECM formation (Gaucherand et al., 2017). Future studies will further elucidate this mechanism and use this knowledge to leverage the potential development of new regenerative therapeutics for dermal wound repair.

## Conclusion

**FIGURE 4:**
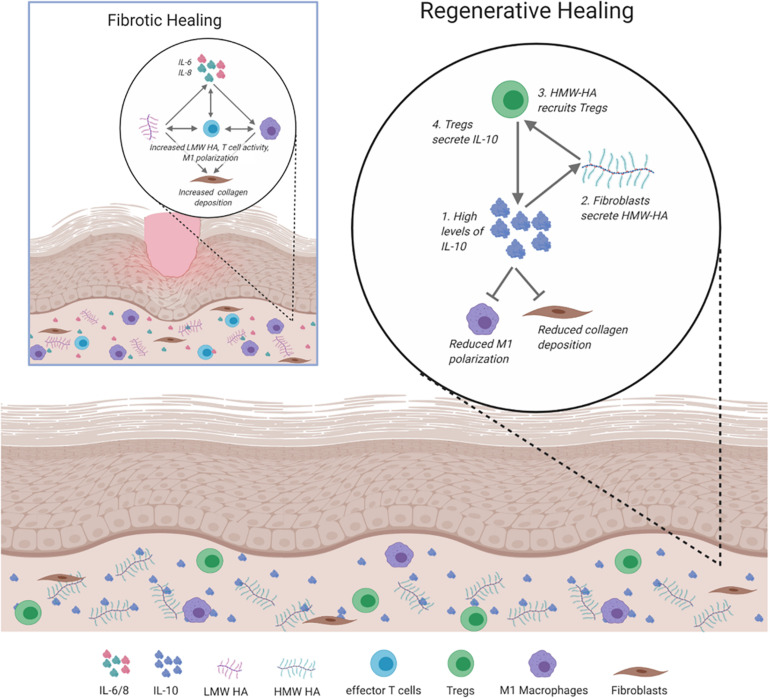
Signaling and cellular regulation pathways involved in fibrotic and regenerative healing. In fibrotic healing, inflammatory signals, including IL-6 and IL-8 activate M1 macrophages to propagate the inflammatory response, activate effector T cells, and recruit fibroblasts to deposit collagen. This is supported by low molecular weight (LMW) HA. Regenerative healing is characterized by reduced inflammation due to the presence of IL-10, HMW HA, and Tregs. IL-10 induces fibroblasts to secrete HMW HA, which results in the increased presence of Tregs and reduced inflammatory macrophage polarization and collagen deposition.

The complex interplay between the inflammatory responses, ECM composition, and immunity is inherently intertwined in directing how skin wounds heal. The key players that balance pro- or anti-fibrotic responses to injury are summarized in Figure 4.

Understanding the framework of mechanisms that coordinate cellular and molecular interactions to result in regenerative or fibrotic wound healing is critical to inform innovative approaches to tissue repair. Ultimately, this knowledge can be leveraged to design and develop next-generation therapeutics to reduce fibrotic responses, and potentially restore the complete integrity of the damaged skin. Initial studies have been performed in humans with local doses of IL-10 at the site of the wounds, leading to reduced fibrosis (Kieran et al., 2013). Similar results have also been obtained in animal models treated with HA and in hypertrophic scars of humans after failed corticosteroid treatments (Mustoe et al., 2002; Hu et al., 2003). The outcomes of ongoing therapies, in addition to studies on fibrosis, underscore the contribution of different cell types, cytokines, and environmental conditions, which include feedback loops between IL-10, HMW HA, and CD4^+^ lymphocytes. Consideration of all of these factors will be essential to successfully treat or prevent fibrosis and change clinical practice.

## Author Contributions

SK conceptualized the review. KS drafted the article. All authors discussed the results, and critically edited the article. All authors contributed to the article and approved the submitted version.

## Conflict of Interest

RB was employed by the company BIOLIFE4D. The remaining authors declare that the research was conducted in the absence of any commercial or financial relationships that could be construed as a potential conflict of interest.
